# Splenectomy in Lymphoproliferative Disorders: A Single Eastern European Center Experience

**DOI:** 10.3390/medicina56010012

**Published:** 2019-12-27

**Authors:** Minodora Onisâi, Ana-Maria Vlădăreanu, Adriana Nica, Andreea Spînu, Mihaela Găman, Horia Bumbea, Irina Voican, Iuliana Iordan, Adrian Alexandru, Mihnea Zdrenghea, Daniela Gheorghita, Sebastian Grădinaru

**Affiliations:** 1 Faculty of General Medicine, University of Medicine and Pharmacy Carol Davila Bucharest, 050474 Bucharest, Romania; minodorel@yahoo.com (M.O.); anamariavladareanu@yahoo.com (A.-M.V.); adriana.nica@suub.ro (A.N.); andreea_spinu09@yahoo.com (A.S.); mihaela_dervesteanu@yahoo.com (M.G.); horiabum@gmail.com (H.B.); dr.adrianalexandru@yahoo.com (A.A.); gradinarusebastian@gmail.com (S.G.); 2 Hematology Department, Emergency University Hospital Bucharest, 050098 Bucharest, Romania; voicanirina@yahoo.com (I.V.); iulia.iordan@yahoo.com (I.I.); 3 Intensive Care Unit, Emergency University Hospital Bucharest, 050098 Bucharest, Romania; 4 Plastic Reconstructive Surgery Department, Emergency University Hospital Bucharest, 050098 Bucharest, Romania; 5 Department of Hematology, Ion Chiricuta Oncology Institute, Iuliu Hatieganu University of Medicine and Pharmacy, 400015 Cluj Napoca, Romania; m.zdrenghea@yahoo.com; 6 Faculty of Materials Science and Engineering, Politehnica University of Bucharest, 060042 Bucharest, Romania; 7 4th Surgery Department, Emergency University Hospital Bucharest, 050098 Bucharest, Romania

**Keywords:** splenic lymphoma, splenectomy, marginal zone lymphoma

## Abstract

*Background and Objectives:* Hematological malignancies are usually systemic diseases of life-threatening impact, and frequently require prompt and energetic therapeutic intervention. Due to systemic involvement, the role of surgery is generally limited to diagnostic approaches, and it is very rarely employed as a therapeutic modality. Splenectomy represents an exception to this paradigm, being used both as a diagnostic and tumor debulking procedure, notably in splenic lymphomas. *Materials and Methods:* We investigated the role of splenectomy in a single center prospective study of splenectomy outcome in patients with splenic involvement in the course of lymphoproliferative disorders. In the present study, we included all patients treated in our department for lymphoid malignancies over a period of six years, who underwent splenectomy as a diagnostic or debulking procedure after referral and workup, or had been referred to our department after first being splenectomized and diagnosed with splenic lymphoma. Patient characteristics and treatment outcome were investigated. *Results*: We enrolled 54 patients, with 34 (63%) splenectomized patients: 12 splenectomies (22.2%) for diagnostic purposes and 22 (40.7%) for treatment. Special attention was given to the 28 (51.85%) patients diagnosed with splenic marginal zone lymphoma (SMZL), a subtype with a clear therapeutic indication for splenectomy. Average age of patients was 57.5 (±13.1) years with a higher prevalence of feminine gender (66.67%). Age above 60 years old (*p* = 0.0295), ECOG (Eastern Cooperative Oncology Group) > 2 (*p* = 0.0402) and B-signs (*p* nonsignificant (NS)) were most frequently found in SMZL patients. Anemia, and notably autoimmune anemia, was more frequent in SMZL versus other small-cell lymphomas and also in splenectomized patients, as was leukocytosis and lymphocytosis. Treatment of patients with lymphoproliferative disorders consisted of chemotherapy and/or splenectomy. Most SMZL patients received chemotherapy as first line treatment (61.5%) and had only partial response (57.7%). Second treatment line was splenectomy in 80% of patients who required treatment, followed by a 60% rate of complete response (CR). Splenectomy offered a higher complete response rate (twice as high than in non-splenectomized, regardless of histology type, *p* = NS), followed by a survival advantage (Overall Survival (OS)~64 versus 59 months, *p* = NS). Particularly, SMZL patients had a 4.8 times higher rate of CR than other non-Hodgkin lymphoma (NHL) patients (*p* = 0.04), a longer progression free survival (73 months vs. 31 months for other small-cell NHLs *p* = NS) and a 1.5fold lower death rate (*p* = NS). The procedure was rather safe, with a 38.5% frequency of adverse reactions, mostly minor and manageable. *Conclusions:* Our data suggest that splenectomy is an effective and safe therapeutic option in patients with lymphoid malignancies and splenic involvement, particularly splenic marginal zone lymphoma.

## 1. Introduction

Hematological cancers are usually systemic diseases, therefore, surgery is very limited as a therapeutic approach. However, surgical biopsies are frequently used for obtaining material for diagnostic pathology examination and various microscopic techniques are used to investigate the biological samples [1,2,3,4]. Splenectomy is a surgical procedure of therapeutic relevance applied in hematology, especially for non-malignant conditions like immune thrombocytopenic purpura where it represents a second line approach. In malignant disorders, splenectomy is performed mainly in primary splenic lymphomas, but also as a debulking procedure in other lymphoproliferative disorders evolving with massive splenomegaly.

Case report studies are very important since they offer possibilities to surgeons from various clinical specialties to be prepared for special situations that appear in their clinical practice [5,6,7].

We aimed to investigate the benefits of splenectomy in patients with chronic lymphoproliferative disorders (CLPD). We monitored the patients who were followed and splenectomized in our hospital, both as therapeutic or as diagnostic procedure–isolated splenomegaly (without adenopathies or other clinical or imaging changes), with or without hematological abnormalities, usually cytopenias, which did not otherwise permit a positive diagnosis.

## 2. Materials and Methods

A prospective observational study was performed between 2010 and 2015, comprising all patients diagnosed with lymphoma and splenectomized who were evaluated in the Hematology Department-University Emergency Hospital Bucharest, Romania. We included 34 cases, of which 22 were splenectomized at our recommendation, and 12 were referred to us after first having been splenectomized and diagnosed with hematological malignancy on pathology examination. Twenty lymphoma patients, non-splenectomized, age-, and gender-matched patients were selected as a control group. Overall, 54 patients with onco-hematological pathology–malignant lymphoproliferative disorders were analyzed for a period of six years.

All subjects gave their informed consent for inclusion before they participated in the study. The study was conducted in accordance with the Declaration of Helsinki, and the protocol was approved by the Ethics Committee of 5218 (Project identification code, approved on 3 March 2015).

Data were obtained directly from the patient as well as from their medical records without interfering with the chosen therapeutic plan. Patient demographics as well as clinical, laboratory, prognostic, and therapeutic parameters were analyzed, as were complications and benefits of splenectomy. The World Health Organization (WHO) classification 2016 was used for establishing the type of lymphoma.

Statistical analysis was performed using EpiInfo version 7 (Centers for Disease Control and Prevention, Atlanta, GA, USA) and GraphPad Software 7 (GraphPad Software, Inc., San Diego, CA, USA). We used non-parametric tests as the studied population did not have a normal distribution. In order to establish risk rates, we computed the odds ratio (OR) and 95% confidence intervals using EpiInfo. Statistical significance was established for *p* < 0.05.

## 3. Results

We enrolled 54 patients with 34 (63%) splenectomized patients; of these, 12 splenectomies (22.2%) were for diagnostic purposes and 22 (40.7%) for treatment. A total of 68.5% had indolent B-cell non-Hodgkin lymphoma (NHL), and 31.5% had aggressive B-cell NHL. Among the patients with indolent NHL, the predominant histological type was splenic marginal zone lymphoma (SMZL) (75.7%), the subtype with a clear therapeutic indication for splenectomy; other subtypes were lymphocytic, mucosa-associated lymphoid tissue (MALT), mantle, and nodal marginal. Of the splenectomized patients, the majority (82.4%) had indolent lymphoma and respectively, 76.4% had SMZL. Therefore, among patients with indolent lymphoma who underwent splenectomy, 92.9% were diagnosed with SMZL (*p* = 0.00005).

The average age of patients was 57.5 (±13.1) years with a higher prevalence of females (66.67%); 44.4% were above 60 years old. Twenty-one patients (38.9%) had an infection with at least one with the hepatitis virus (HBV/HCV) with predominance for HCV–14/21 (66.7%). The prevalence of viral infections in SMZL patients was 4.2% HBV and 14.8% HCV.

The results of the statistical analysis are summarized below and in Table 1 for the most relevant differences. As SMZL patients represented the majority, special attention was given to this subgroup.

The age of the SMZL patients was not statistically different from other indolent lymphoma patients; studies have reported a slightly higher age at diagnosis [8]. However, age over 60 years old (negative prognosis factor in lymphomas [9,10]) was found three times more frequently in SMZL patients versus the rest of the lymphoma patients, *p* = 0.0295. Poor performance status ((Eastern Cooperative Oncology Group) ECOG > 2) was more commonly found among patients with SMZL than in other small-cell NHLs (risk difference 31%, *p* = 0.0402). Additionally, the rate of splenectomy was 21% higher in patients with unfavorable ECOG (<2), *p* = 0.088. Constitutional (B) signs were 2.3 times more frequent in patients with SMZL versus other indolent NHLs (*p* > 0.05), thus conferring SMZL patients with a poorer prognosis. For splenectomized patients, we noticed the same trend, but with lower differences and no statistical significance.

The prevalence of bulky disease (masses larger than 10 cm) was 37.5% higher in SMZL patients versus other indolent NHLs, *p* = 0.005. We found no differences between the splenectomized and non-splenectomized patients. Extranodal involvement was rare in SMZL patients (OR = 0.51, p-NS), as was also seen in splenectomized patients (p-NS). Hypoalbuminemia was slightly more frequent in SMZL versus other indolent NHLs (*p* = NS); however, in splenectomized patients, hypoalbuminemia was significantly more frequent.

**Figure 1:**
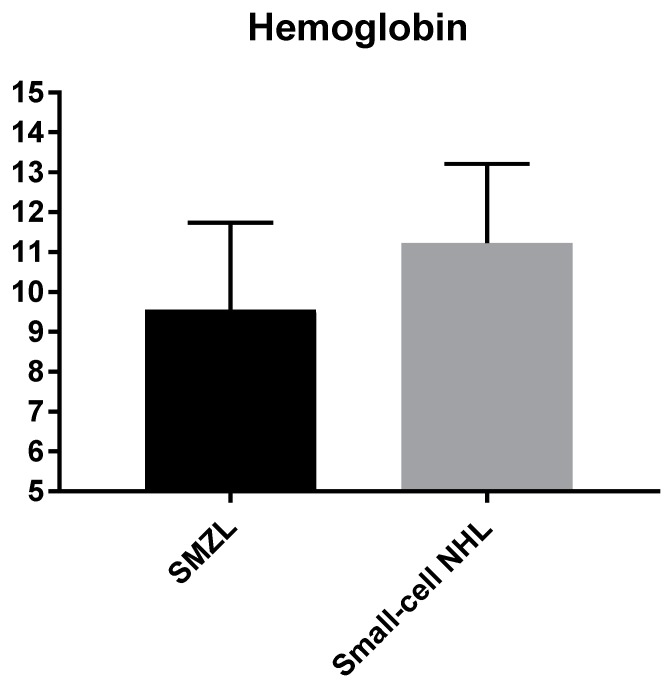
Hemoglobin level measured for splenic marginal zone lymphoma (SMZL) patients and indolent non-Hodgkin lymphoma (NHL).

**Figure 2:**
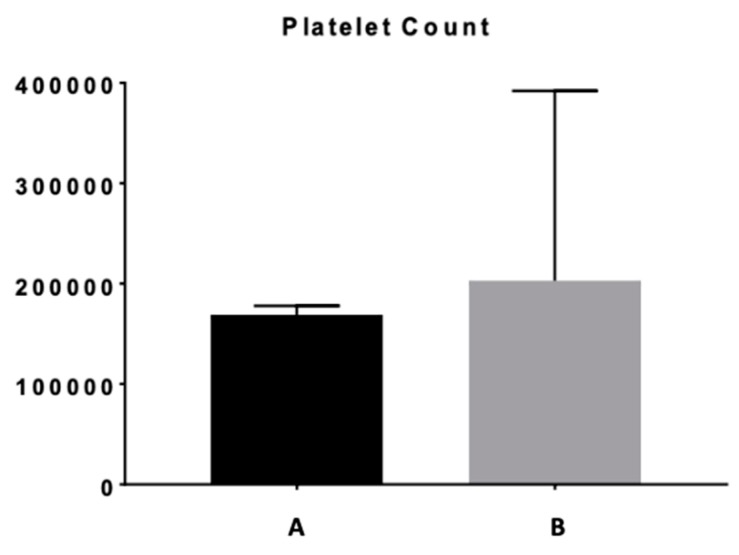
Platelet count for (**A**) SMZL patients and (**B**) indolent NHL.

Analyzing hematological patterns, we observed that patients with SMZL had a supplemental degree of anemia (Table 1, Figure 1) and also of thrombocytopenia (Table 1, Figure 2). We also found that autoimmune anemia had a higher prevalence in SMZL patients than in other indolent NHLs, p-NS; splenectomized patients presented more often autoimmune anemia, with statistical significance (Table 1). Leukocytosis and lymphocytosis were notably more frequent in SMZL and respectively in splenectomized patients (Table 1).

The marrow infiltrate was higher in SMZL patients (35% versus 19% in other indolent NHLs, *p* = NS). Additionally, splenectomized patients had a higher infiltrate irrespective of their type of lymphoma (~27% versus ~18% for non-splenectomized ones, *p* = NS).

Regarding staging at diagnosis (according to Ann-Arbor classification), there were no differences in patients with SMZL versus other lymphomas, but splenectomized patients were more frequently in advanced stage (respectively stage III/IV).

For SMZL patients, we computed the specific prognostic score SMZL IIL (Italian Lymphoma Group) using hemoglobin, albumin, and lactate dehydrogenase (LDH). A total of 76.9% of patients were placed in the high-risk category with two or more risk factors and an estimated 5-year survival of ~56%. We also calculated the SMZL Study Group score, respectively, the specific prognostic index (IP), and almost all patients (88.5%) were high-risk. The treatment options were also analyzed.

Time to therapy initiation was shorter in SMZL patients versus other indolent NHL (1.5 months vs. ~9 months). In patients who underwent splenectomy, therapy was indeed performed much faster, at about 1.6 months after diagnosis compared to four months in non-splenectomized patients.

Chemotherapy protocols included Rituximab (antiCD20 monoclonal antibody) in most SMZL cases (84.2%) and CVP/CHOP protocols (95.5%). For the majority of patients included in this study, the median number of chemotherapy cycles for the first line of treatment was six. It is important to observe that non-splenectomized patients received fewer therapy cycles compared to splenectomized ones (~4.8 vs. ~6.4 cycles, *p* = 0.0051), most likely because splenectomized patients had worse prognosis and more advanced lymphomas, and therefore required a more aggressive therapy.

For splenectomized SMZL patients:

- Response to first-line treatment (mostly chemotherapy-61.5%) was generally partial (57.7% cases). As expected, chemotherapy as a single treatment was not associated with complete response in these patients. Thus, 76.9% (20/26) needed second line therapy, which was splenectomy in 80% of cases. The benefit of this therapeutic option was very good, because there was a 60% complete response (CR) rate.
- Splenectomy represented the first line treatment for only 34.6% of cases.
- 38.5% of patients presented different side effects after splenectomy (*n* = 26). Most of these were thrombotic complications in the abdominal area (four cases), followed by infections (approximately 30%), mechanical complications (eventration, hernia), and very rarely bleeding (accounting for 10% of all complications after splenectomy, respectively 3.8% of splenectomized patients).

Regarding the benefit and treatment response, the data confirmed that the CR rate in the SMZL group was significantly (*p* = 0.04) higher (about 4.8 times higher) than in other indolent NHL patients, and that the CR rate in the splenectomized patients was twice as high as that in the non-splenectomized, regardless of histology type (*p* > 0.05). In SMZL patients, CR was 17% more frequent if chemotherapy was applied after splenectomy (usually when progressive disease occurred), compared with the CR rate for SMZL patients treated first with chemotherapy, followed by splenectomy in second line treatment.

The death rate in SMZL patients was lower when compared to the other indolent NHL patients, about 1.5-fold (*p* > 0.05), with a clear benefit of combination therapy in SMZL patients (chemotherapy-splenectomy or splenectomy-chemotherapy). Additionally, splenectomized NHL patients, regardless of histology type, had an overall death rate lower than non-splenectomized patients of ~2.8 times (regardless of the cause of death, but more often due to the associated hematologic neoplasm), *p* = 0.06.

**Figure 3:**
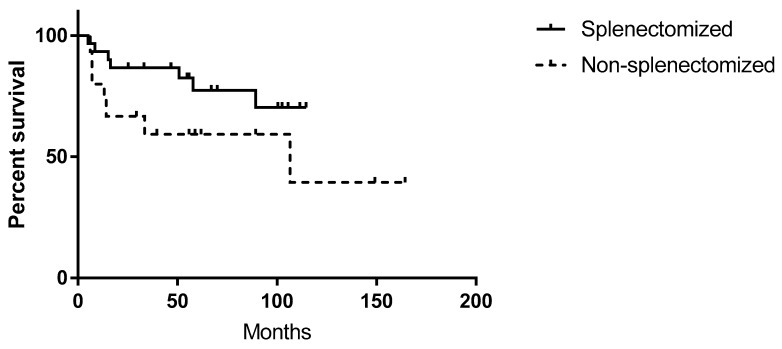
Kaplan–Meier survival curves in splenectomized and non-splenectomized patients.

Overall survival (OS) (Figure 3) was higher in splenectomized patients (64.48 ± 33.21 months versus 59.19 ± 44.78), although not statistically significant (*p* > 0.05).

The relapse rate (for patients who achieved response) was lower in SMZL compared to the rest of the indolent non-Hodgkin lymphomas patients, about 1.4 times, *p* > 0.05. Additionally, the relapse rate was reduced for NHL patients who underwent splenectomy compared to the non-splenectomized ones, about 1.5 times, *p* > 0.05. After splenectomy, SMZL patients generally did not relapse (60.9%).

Regarding progression free survival (PFS), there was a clear benefit in the SMZL group compared to indolent NHL patients (about 2.5-fold), 73 months versus 31 months, *p* > 0.05. Data were similar for splenectomized patients with a PFS almost 2.3 times higher for splenectomized cases than for non-splenectomized patients, *p* > 0.05.

Transformation rate to a more aggressive disease, usually non-Hodgkin large cell lymphoma histology type, was 1.4-fold higher for SMZL patients, *p* > 0.05. This is probably the explication for the lack of survival advantage in SMZL, although the CR rate was obviously higher and the relapse rate was lower in these patients.

In addition, splenectomy induced longer disease free survival versus other treatment options (121 months vs. 13 months, *p* > 0.05 due to reduced patient numbers).

## 4. Discussion

Although splenic involvement can occur in any type of lymphoma, it is mostly seen in indolent/low-grade non-Hodgkin lymphomas (NHLs)/chronic lymphoproliferative disorders (CLPDs) like marginal zone and follicular lymphomas, hairy cell leukemia, and chronic lymphocytic leukemia/small lymphocytic lymphoma. Marginal zone lymphomas (MZLs) are a group of indolent B cell CLPDs, representing about 10% of all adult lymphomas; these are generated by the proliferation of malignant cells with a normal counterpart in B lymphocytes present in a particular peripheral area of the lymphoid follicle, the marginal zone [11]. MZL is further sub-classified as splenic marginal zone lymphoma (SMZL), usually presenting with massive isolated splenomegaly, nodal MZL, and extranodal MZL MALT type [12].

SMZL is a rare indolent neoplasia. Patients typically have abdominal manifestations including splenomegaly, sometimes liver involvement and mesenteric or splenic adenopathies (without peripheral adenopathy) and may consequently present with abdominal symptomatology. Bone marrow and peripheral blood involvement are frequent, which allows for a positive diagnosis by immunophenotyping of peripheral lymphocytes [13]. Serum or urinary monoclonal peaks are present in 30% of cases. It may be associated with chronic hepatitis C virus infection, and antiviral treatment is warranted in suitable patients. For the remainder, recommended first line approaches are splenectomy or rituximab immunotherapy, sometimes accompanied by chemotherapy [14].

Splenectomy in hematological patients has low rates of morbidity and mortality (of around 12% and 1%, respectively) [15]. In SMZL, retrospective studies have shown improvement in symptomatology and cytopenias after splenectomy, which persist up to five years after splenectomy [16,17,18,19]. Eventually, residual disease in the bone marrow will lead to progression. Improved overall survival in splenectomized patients over those treated with chemotherapy (without rituximab) has been reported [20].

In view of the literature data, we observed the characteristics and evolution of splenectomized patients with CLPD. As expected, the majority had indolent lymphoma, namely SMZL (28/54 = 51.8% of all patients, respectively 76.4% of splenectomized patients).

Among the SMZL patients, a higher occurrence of negative prognostic factors has been discovered: age over 60 years, constitutional signs, hypoalbuminemia, ECOG > 2, and bulky disease, as reported elsewhere [9,10,13,20,21,22]. Thus, most of the SMZL patients were high-risk, with an estimated 5-year survival between 44–69% [17].

Prevalence of hepatitis viral infections in SMZL patients was concordant with other studies, showing an association between HCV infection and SMZL [20,23,24].

Splenectomized patients were found more frequently with B signs, unfavorable ECOG, and hypoalbuminemia, and thus we can uphold that splenectomy was not at all restricted to patients with good clinical status, but actually it was more frequently offered to patients with negative prognostic factors and poorer clinical status. It is also noteworthy that splenectomized patients were more frequently in an advanced stage of disease, confirming the actual benefit of this therapy, which was chosen and successfully performed in patients with more severe disease.

For disease behavior, we found no differences regarding bulky or extranodal involvement, as expected, since patients who underwent splenectomy irrespective of their disorder were less likely to have extranodal involvement: splenectomy was performed for diagnostic purposes (so no other organs were affected) or therapy (the spleen was the main site affected and the surgery accomplished a significant or complete tumor reduction).

The degree of anemia and thrombocytopenia was higher in SMZL patients; this was likely linked to the splenomegaly and inherent hypersplenism, as platelets are most likely to be pooled in the large spleen [25]. The observation is also valid for splenectomized patients, but in this case, a significant contribution also goes to the more advanced stage of disease of these patients. 

Autoimmune anemia was found to have a higher prevalence in SMZL patients (*p* = NS) concordant with the literature [20,26,27,28] and represents a significant contribution to the higher degree of anemia of these patients. Additionally, splenectomized patients presented more often with autoimmune anemia (*p* = 0.027); and, because splenectomy is also effective therapy for this specific complication [29], it represents a very helpful therapeutic tool for a particular subgroup of patients, not only for the treatment of malignant pathology but also for the associated complication, autoimmune anemia.

The median white blood count (WBC) was higher in SMZL patients (although p-NS). Interestingly, splenectomized patients also had a clearly higher WBC than non-splenectomized ones (*p* = 0.05), probably due to the high frequency of SMZL patients among the splenectomized ones; also, splenectomy was performed as a therapeutic option in order to reduce the high tumor mass also reflected in the leukocytosis.

We also studied the presence of lymphocytosis in order to explain the leukocytosis and to prove that the higher WBC was related to the hematological condition and not to other associated problems. We observed a six times higher rate in SMZL versus other indolent lymphomas (*p* = 0.018) due to the marrow involvement (see below). Moreover, splenectomized patients had a nine times higher rate of lymphocytosis versus non-splenectomized patients (*p* = 0.0004), thus demonstrating the major benefit of surgery that was chosen as therapy in malignant hematological disorders with very high tumor burden and peripheral involvement.

The marrow infiltrate was higher in SMZL patients, as reported in the literature: SMZL is usually diagnosed in stage IV, but also keeps the indication for splenectomy as first line treatment [20]. This also concords with the observations above regarding more frequent cytopenias in SMZL (anemia, thrombocytopenia) and more frequent leukocytosis and lymphocytosis, all due to higher marrow lymphoid infiltrate. Additionally, splenectomized patients had a higher infiltrate irrespective of their type of lymphoma. We can thus conclude, as other authors have proven [30], that splenectomy is a very valuable therapeutic instrument even though the disease is clearly not limited to this organ (and therefore it is not a curative intent), since it offers a marked tumor reduction and offers the patient a plateau, a period of time free from treatment (chemotherapy), and even offers the possibility of treatment for associated complications such as autoimmune anemia.

Considering that in the studied group, splenectomy was more frequently offered to patients with more reserved prognosis, more advanced stage and ECOG as well as worsened hematological features, we analyzed the evolution after treatment.

SMZL patients received mostly chemotherapy as the first line treatment and only in one third of the cases was splenectomy chosen as the primary therapy. As a result, most of them required a second line treatment, represented by splenectomy, subsequently obtaining a high rate of complete responses, respectively 4.8 times higher than the CR rate in other small-cell NHLs. Interestingly, the CR rate in SMZL patients was higher if splenectomy was first line versus second line. Although not statistically significant, the observation is relevant and underlines once again the benefit of splenectomy in SMZL patients; the literature data show indeed that splenectomy is recommended as the first line treatment option and is associated with a factual survival benefit [16,18,19,30,31,32].

Splenectomy offered indeed a longer PFS (with higher complete response rate) in NHL patients; it also had a lower death rate and a superior overall survival versus non-splenectomized patients, consistent with the literature that shows a higher overall survival in patients who underwent splenectomy [16,17,18,19,30,32]. However, one should take under advisement the fact that most of the enrolled patients had indolent lymphomas, with a clear predominance of SMZL.

In our group, splenectomy was found to be beneficial, being associated with higher rates of complete responses and improved survival as well as lower rate of relapses and higher progression- and relapse-free survival.

The procedure was rather safe, with only a slightly higher rate of complications than previously reported [15,33,34,35], mostly thrombotic as explained by the thrombotic risk associated with any surgical procedure; by the physiological thrombocytosis secondary to splenectomy; and the additional risk factors of this procedure performed in patients with an active neoplasm, usually with a median age over 60 years old, as mentioned before.

Finally, we would like to mention some limitations of this study:

- The number of enrolled patients was low;
- We selected a control group of patients with NHL (non-splenectomized) to match the splenectomized ones as close as possible, before splenectomy took place. There might be a selection bias, as the discussed pathology is rare, and the study comprises only one center, which is located in a large emergency hospital in the capital city, receiving all types of hematological disorders; furthermore, splenectomy is not a frequently used procedure in malignant hematology.

## 5. Conclusions

We found that in lymphoid neoplasms with splenic involvement, namely SMZL, splenectomy represents an extremely valuable and useful treatment option, even in patients with advanced stages, poor prognostic factors, or complications like autoimmune hemolytic anemia. The benefit of splenectomy was demonstrated by a higher rate of complete responses, lower rate of relapses, and longer disease-free survival, thus offering a survival advantage. Our study endorses that, in the era of rapidly accumulating new therapeutic options, traditional approaches like splenectomy should be taken into account in this group of patients.

## Figures and Tables

**Table 1:** Laboratory evaluation of the studied patients.

Parameter	Lot	Value	*p*
Age (years)	SMZL Indolent NHL	59.5 ± 9.7 61.5 ± 10.4	NS
	Splenectomized Non-splenectomized	59.0 ± 9.5 55.1 ± 17.6	NS
Hemoglobin (g/dL)	SMZL Indolent NHL	9.5 ± 2.1 11.2 ± 1.9	0.049
	Splenectomized Non-splenectomized	10.3 ± 2.2 10.5 ± 2.6	NS
Leucocytes (× 10^3^/mmc)	SMZL Indolent NHL	18.3 ± 16.8 9.3 ± 4.2	NS
	Splenectomized Non-splenectomized	16.9 ± 15.5 8.6+ ± 4.1	0.050
Thrombocytes (× 10^3^/mmc)	SMZL Indolent NHL	172.2 ± −97.0 270.7 ± 133.0	0.019
		**Risk rates (OR)**	
Lymphocytosis	SMZL Indolent NHL	6.00	0.018
	Splenectomized Non-splenectomized	9.15	0.0004
Low albumin	SMZL Indolent NHL	1.73	NS
	Splenectomized Non-splenectomized	3.15	0.040
Autoimmune anemia	SMZL Indolent NHL	3.78	NS
	Splenectomized Non-splenectomized	6.84	0.027
Advanced vs. early stage (Ann-Arbor)	SMZL Indolent NHL	NS	NS
	Splenectomized Non-splenectomized	2.58	NS

SMZL = splenic marginal zone lymphoma, NHL = non-Hodgkin lymphoma, NS = nonsignificant.
